# The Effect of Cyberpower on Institutional Development in Norway

**DOI:** 10.3389/fpsyg.2018.00717

**Published:** 2018-05-15

**Authors:** Benjamin J. Knox

**Affiliations:** ^1^Norwegian Defence Cyber Academy, Lillehammer, Norway; ^2^Department of Information Security and Communication Technology, Norwegian University of Science and Technology, Gjøvik, Norway

**Keywords:** cyberpower, institutional development, governance, sector-principle, cyber-security, Norway

## Abstract

Through analysis of empirical interview data this research undertakes to investigate the ways in which the growing phenomenon of cyberpower – defined as using cyberspace for advantage and influence – is impacting on institutional development in Norway. Exploring this governance challenge through the conceptual framework of complexity, difference and emergence opens space – political or otherwise – for discussion regarding why rapid developments arising from digitalization are transforming the way individuals, organizations, institutions and states behave, relate and make decisions. Cyberpower is creating an uncertain institutional landscape as a dependency vs. vulnerability paradox shapes values, rules and norms. Findings from this thematic analysis of qualitative data reflect this paradox, and suggest that organizations in Norway are in a survival-mode that is blocking collaboration. This occurs as national governance systems, human capacity and cyberpower effects lack synergy making for an uneasy arena where complexity, contestation and emerging challenges frame institutional development. To improve long-term prospects of governing cyberpower effects requires a cross-sectorial conflation of time and human resources. This means consciously taking steps to merge organizational and institutional boundaries through expressive innovative collaborations that foster a shared and holistic agenda. The emerging challenges cyberpower is presenting across multiple domains means further research is recommended to build a richer understanding of the term cyberpower from different perspectives. The investigation recommends investment in building the skills and capacities necessary for the co-creation of new models and strategies for managing the effects of cyberpower.

## Introduction

Improving cyber security prospects, nationally and internationally, involves key institutions taking the greater share of responsibility in deciding how to govern the growing influence of power being exercised through cyberspace (Tapscott, 2014; Hagen, 2016; Norwegian Centre for Information Security [NorSIS], 2016). In real world terms, this emphasizes the importance of shared responsibility (Thomas, 1996) when managing powerful actions that occur through cyberspace, such as those that threaten democracy itself (Vatu, 2017). Despite this, cyber security is largely: “…controlled by the private sector and other nonstate actors” (Peña-López, 2016, p. 223). Therefore negotiating and brokering this fragmentation to bring together those with complentary needs (Thomas, 1996) becomes a governance challenge. This study takes a cross-sectorial approach aiming to build knowledge by identifying how different organizations within seven key sectors, all of whom are notable cyberpower stakeholders, in Norway (political, military, economic, social, informational, infrastructure and diplomatic) manage increased levels of uncertainty arising from complex and emerging challenges presented by cyberpower effects.

The Norwegian state is governed through a national sector-principle framework. This strategy was formally implemented in the 70s but has been practiced since the 1800s. Each Government Ministry is highly autonomous and specialized within its own domain. By encouraging sector-orientated development, ministries are empowered and responsible for their own policy formulation and implementation. From a critical perspective this has been described as creating segmentation within the state (Egeberg et al., 1978) resulting from poor cross-sectorial coordination and selective action (Organisation for Economic Co-operation and Development [OECD], 2005). However, the culture of skepticism toward centralization of power – retained from days of Swedish and Danish rule – means the sector principle takes precedence. To ease tensions that can emerge between centralized and cross-sector power dynamics, inter-sectorial relationships are encouraged beyond ministerial level and are expected to work on the assumption that they are based upon trust, shared values and goals; when managing threats, challenges and vulnerabilities to systems of shared national interest. Recently, in light of the 22 July 2011 terror attacks in Oslo as well as threats presented through cyberspace (Meld. St. 10, 2016–2017); such as a significant increases in cybercrime and targeted cyber-operations by Russian hackers on Norwegian defense and security officials (The Guardian, 2017); the government has been required to take a more central role in for example, national crises management (Gjørv, 2012).

Given their power and autonomy, sectors have institutionalized certain cyberpower capacities particular to their individual needs and goals. For example military use of sensor capabilities in support of national and international computer network defense; the application of advanced software to remain competitive in global financial markets; information operations to project a national narrative through digital media outlets; or the utilization of digital command and control technologies for increased efficiency in the energy and diplomatic sectors. Whatever the capacity, it is essential to understand that, similar to other well-intentioned institutionalized practices, advantages are often undermined by related vulnerabilities. In this case, dependency and reliance upon cyberspace provides opportunities for opponents with cyberpower capacities to also: “…use cyberspace to create advantages and influence events […] across the instruments of power” (Kuehl et al., 2009, p. 38). This dynamic is redefining institutional rules of the game in society (North, 1990; Tapscott, 2014). In developmental terms, cyberpower has the capacity to bring about “good change” (Chambers, 1995, p. 174) by providing opportunities for expanded agency leading to innovative collaborations. For example the rise of inter-enterprise computing allows for the blurring of inter-organizational boundaries (Ostrom, 1996) enabling new relationships to emerge that transform business models and strategies (Tapscott, 2014). Entrepreneurship can now reach far beyond physical borders due to globally integrated markets and rapidly expanding networks unhindered by time zones (Castells, 2011). Simultaneously though, cyberspace gives agency and opportunity to antagonists and criminals to challenge and undermine systems of governance, coordination, cooperation and competition within and beyond cyberspace (Nye, 2011). In 2014 the global costs of cybercrime was estimated between US$375 billion and US$575 billion, that’s 0.6 percent of global GDP (Center for Strategic and International Studies and McAfee [CSIS], 2014, p. 2). The same report puts Norway’s loss as 0.64% of national GDP (p. 9). Norwegian GDP in 2014 was US$498 billion (World Bank, 2017, online), which equates to a loss of US$3.2 billion.

Cyber security can be understood as attempting to protect cyberspace and those tangible and intangible assets that function within it: “…relating to the wellbeing of either individual or society at large” (von Solms and van Niekerk, 2013, p. 101). The Norwegian state takes a multi-stakeholder approach (Chehadé, 2014) to cyber security. According to Muller (2016) the multi-stakeholder model is an extension of neo-liberalist thinking as the intention is to achieve streamlined Internet governance through decentralization of responsibility and power to promote cooperation between the state, private sector and civil society. Although this approach is generally seen internationally as best practice for cyber security (Carr, 2015), even in Norway, where public-private trust relationships are deemed to be good, there are tensions arising from established power dynamics between actors, as those stakeholders deemed to be the most appropriate for good governance have so far failed to come together to address the key challenges and vulnerabilities presented through cyberspace (Helkala and Svendsen, 2014; Muller, 2016).

The fact that the Norwegian government allows sectors to develop with relative autonomy makes it important to understand cyberpowers influence on how institutional development processes associated with cyberspace operate across sectorial domains. The typology of this rapidly developing phenomenon mean appreciating motivations and conflicts related to value-based decisions – that frame selective actions and implementation of protocols intended to prevent abuses of cyberpower – become crucial if trust, holistic approaches and collaborations are to form the foundations of development actions (Hartley et al., 2002; Tapscott, 2014). This is corroborated by earlier research that highlighted key national challenges relating to cyber governance across government institutions; and a lack of inter-organizational coordination and public-private partnerships leave uncertainty concerning implementation of: “…international standards, recommendations and best practices into national strategies and guidelines” (Helkala and Svendsen, 2014, p. 10).

Multiple complex processes and interactions occur at the interface of institutional, organizational and individual development (Hartley et al., 2002; Leftwich and Sen, 2011). When interfaces are less visible and shifting in a context of value-based conflicts it is important to learn from different perspectives and understand how competing processes create tensions, due to inappropriate structures or inefficient practices. Deeper appreciation for, and understanding of the institutional landscape can support better governance action capable of coping with and responding to: “tensions, conflict and (re) negotiation” (Wuyts, 1992, p. 280).

In this study, the complex issues around the governance of cyberpower will be treated as emerging and increasingly dominant orthodoxies that are disrupting established patterns of living (Thomas, 2000). By deductive analysis of empirical interview data (Elo and Kyngäs, 2008) this research explores perspectives from key organizations within seven Norwegian sectors; this research seeks to deepen understanding and inform for greater appreciation of the discourse convergence and divergence involving multiple stakeholders, disciplines and governance levels in society, that all contribute to an institutional landscape that is being shaped by a new form of power. The State of Art for this paper is structured in a Complexity, Difference, and Emergence conceptual frame. The Section “Methodology” describes the qualitative research approach and design. The Results are categorized and presented thematically. Further, the Section “Discussion” frames key findings in the wider problem context. The Section “Conclusion” makes a number of concrete recommendations as well as plan for Section “Future Work” to add depth and validity to this initial work.

## State of Art

Around the world, governments of both developed and developing economies are taking action at national level to address cyber security concerns […] there are few obvious policy recommendations…

(Peña-López, 2016, p. 223)

In Norway, the unevenness between sectors, i.e., their relative autonomy – represents institutional contradictions that can lead to unintended consequences (Engberg-Pedersen, 1997) as processes of divergence and convergence occur as reactive/un-planned policy, practice, and governance responses to emerging challenges.

The CoDE conceptual framework (Pinder, 2016) is a highly relevant device for opening up and deriving insights into the ways in which cyberpower penetrates across ***Co****mplex* institutional landscapes – in Norway as well as globally – characterized by multiple interested parties, multiple fields and multiple levels of governance. Appreciating how the ***D****ifference* in organizational identities and understandings might affect institutional development processes as each sector comes with its own interests, values, agendas, and culture may help to explain why cyberpower – as an ***E****merging* and dynamic governance problem, characterized by uncertainty and unpredictable outcomes – is difficult to grasp beyond military applications.

The independent and overlapping elements of the CoDE framework can help to explain an environment where the effects of cyberpower are delivering impacts upon social change processes that cannot be known in advance (Thomas, 1996). The CoDE framework implies that institutional development processes, taking place in over-lapping spheres (Leftwich, 1996) occur in a multitude of ways and that, given the trans-boundary nature of the cyberpower phenomenon, situating organizations in their national institutional context may not necessarily define: “what is deemed possible, acceptable and legitimate” (Hartley et al., 2002, p. 394). Instead there are competing paradigms as values, rules and norms (Brett, 2000) within one institution may not be compatible with emerging institutional characteristics of another. One outcome of this could be constrained action, as the effects adversely impact organizational processes at the social and cultural system levels (Hartley et al., 2002). This reinforces the importance of building connections between institutions through cooperation – that may require forms of interagency collaboration (The Open University, 2000) – and vertical and horizontal co-ordination. It also shows how institutions: “…by producing stable, shared and commonly understood patterns of behavior, are crucial to solving the problems of collective action amongst individuals” (Brett, 2000, p. 18).

### Complexity

From a development management perspective, it is understood that institutions provide the frameworks within which organizations operate. Similarly: “effective organizations depend on the existence of institutions which constitute the rules which everyone – including the managers – must accept” (Brett, 2000, p. 19). This builds on the idea that institutional change cannot be achieved by a single agency with control over resources and processes (Thomas, 1996). This was echoed in a recent Norwegian Government document that specified that a direct consequence of being one of the world’s most digitized nations is the level of vulnerability that digital dependency brings, and dealing with this operational problem at sector or national level cannot be managed by one single agency (Stortingmelding, 2016–2017).

Authors such as Nye (2011) and Jasper (2012) identify the state as the balancing agent when complex tensions arise from the dependency vs. vulnerability paradox in conditions where: “The more advanced a nation becomes, the more it relies on access to the commons and the more vulnerable it becomes to the loss of access” (Jasper, 2012, p. 60). This captures the Norwegian context in relation to the complex implications of cyberpower and how effects can influence institutional development across domains. Norwegian researchers and cyber security experts have called for greater governmental responsibility and control in securing a cross-sectorial approach (Helkala and Svendsen, 2014; Norwegian Centre for Information Security [NorSIS], 2016). For example analysis of Norway’s cyber and information security strategy identified three levels of potential target; national, organizational, and individual. Each level has assets to protect, but irrespective of criticality, each is vulnerable to nefarious actors using cyberpower to target them. In a context of digital dependency, the study identified a lack of know-how and functioning legal frameworks across multiple fields within which to manage the positive and negative effects of cyberpower (Helkala and Svendsen, 2014). This raises management questions when sectors have designed, developed, and directed independent cyber security solutions, meaning vulnerabilities and dependencies are embedded in cultural collectives and institutions (Crewe and Axelby, 2013) that span multiple interested parties, multiple fields and multiple levels.

In Long’s (2001) definition of power he states how power is: “the outcome of complex struggles and negotiations […] and necessitates the enrolment of networks of actors and constituencies” (p. 71). When we consider that the real world effects of cyberpower are only emerging through an ever expanding and entangled digital network of known and unknown actors, then the outcomes, or the potential of power exercised through cyberspace: “…clashes with our habitual patterns of the classification of things” (Betz and Stevens, 2011, p. 128) on the basis of authority, status, and reputation. Threats to any one of these can present institutional and capacity constraints to the effective functioning of inter-organizational relationships and policies on many levels, and across sectorial fields (Kickert et al., 1997).

Working for effective collaboration – the type that creates collaborative advantage for broader social objectives (Huxham, 1993) – between different organizations becomes a complex struggle as each one independently seeks to manage tensions created by various forms of power delivered through cyberspace. This reveals how cyberpower, in line with other dimensions of power is relational, its struggles lie at the heart of institutional development and it needs to be interpreted in the complex context in which it is being conveyed (Rowlands, 1997; van Haaster, 2016).

### Difference

We can no longer take for granted what Thomas (1996) wrote about in terms of understanding development as a: “…long-term process of social change” (p. 98) or that it: “cannot be controlled by human agency” (p. 97). What we are witnessing today is human agency empowered by cyberpower influencing and driving social change at rates traditional good governance systems, and codes of practice cannot control (Stevens, 2015). When state frameworks are unable to manage dynamics central to multi actor steering, such as communication and control (Rosenau, 1995); then good purposive governance is challenged. From a global macro level we are witnessing technological advancements outpacing and out performing human agency (Castells, 2011). Simultaneously at state level – as Norway is experiencing – there are concerns regarding how to manage and govern consequences of cyberpower, such as tensions arising from the aforementioned digital dependency vs. digital vulnerability paradox.

When the rules of the game are changing (North, 1990) and tensions lead to: “imperfect compromises” (Nye, 2011, p. 16), then managing and operating in this complexity may require greater appreciation of the digital environment and the space it opens up for cyberpower to influence institutional landscapes. These conditions reflect the idea that understanding where relative power lies and identifying room for maneuver may be more important than specific skills (Thomas, 1996). A national study into Norwegian cyber security stated that appreciating cyber security culture: “…touches upon some of the most profound questions for development” (Norwegian Centre for Information Security [NorSIS], 2016, p. 13). For some, technology and its power can: “…take us into an age of barbarism that will make fascism look like an exercise in charity and human progress (Courneyeur, in Tapscott, 2014, p. 387); for others, it creates a new environment for collaborating on shared interests (Tapscott, 2014). These polarized perspectives resonate with the idea of: “bringing together those with complementary needs” (Thomas, 1996, p. 107) and presents the opportunity to move governance approaches and thinking to: “conceptualizing a whole development management arena as an inter-organizational domain” (ibid, p. 107).

It is generally accepted that best practice for mitigating negative effects and leveraging positive effects of cyberpower is for organizations to adopt a holistic approach (Castells, 2011; Nye, 2011; Jasper, 2012; Tapscott, 2014; Hagen, 2016; Norwegian Centre for Information Security [NorSIS], 2016). Inevitably though, rather like evaluating good practice (Everitt, 1996), agreeing on a best practice is contested. If organizations are to avoid collaborative pitfalls arising from known and unknown vulnerabilities (Huxham, 1993); then a holistic approach will require negotiation of values, goals, interests and meaning agendas between organizations prior to any formal or informal process of co-ordination/co-operation or collaboration occurring.

The key to achieving the profits of digitalization is: “collaboration and openness” (Peña-López, 2016, p. 223). Implicit in collaboration are the functions of negotiation, information sharing, and transparency (Robinson et al., 2000). As valuable as these concepts are for institutional development, they generate feelings of vulnerability at a personal and organizational level, as they demand the giving up of power for progress. In a development landscape with no clear architecture or governance systems, attitudes to collaboration can harden, pushing organizations apart as they are less willing to embrace collaborative opportunities (Turkle, 2011; Norwegian Centre for Information Security [NorSIS], 2016); due to the value-based conflict between them and the transaction costs (Ouchi, 1980) involved in collaboration. Even so, these functions are seen as necessary for implementing holistic approaches to managing the effects of cyberpower (Thomas, 1996; Nye, 2011; Castells, 2011; Tapscott, 2014).

There will come a point when the requirement for inter-organizational negotiation becomes real and organizational collaboration needs to aspire to more than just a token concept (Huxham, 1993). This assumption is drawn from the idea that: “Before embarking on a strategy of coordination it is important to check that the potential for collaboration exists” (The Open University, 2007, p. 70) among different stakeholders. In this context, aligning collaboration with coordination and cooperation, will see collaboration going beyond its role of achieving influence in public action (Thomas et al., 2001), to one of adding a deeper level of understanding concerning how might cyberpower be driving inter-organizational behavior.

In an uncertain or shifting landscape (Hartley et al., 2002) understanding management approaches across critical sectors may reveal organizational blind spots, blurred boundaries, and how different organizational types are responding to emerging cyberpower effects in contexts characterized by value based conflicts and multiple competing actors (Thomas, 1996). Aligning governance frameworks with real world emerging cyberpower effects driving institutional development, can build inter-organizational relationships that support collaboration within a digital society.

### Emergence

Considering the level of digital resource dependency in Norway, one could assume that the state backed sector-principle and a multi-stakeholder approach to cyber security forms a solid: “…basis for organizational linkages” (Salancik and Pfeffer, 1978; in Thomas, 1996, p. 107). In this context, multiple stakeholders have a part to play in managing factors that impinge on Norway establishing: “the necessary preconditions” (Muller, 2016, p. 2) for protecting assets from vulnerabilities presented through cyberspace. This is critically important as it reveals that environments – framed by cyberpower – are emerging and presenting interesting challenges to theories and practice of governance. Most significant is the idea, referred to above, of development and its management as being a shared responsibility (Thomas, 1996).

Learning from the Norwegian context may have wider applications. For example Crewe and Axelby (2013) discuss the idea of time following linear anthropological steps in modernity theory. This idea is challenged as Internet access becomes ubiquitous and the North and South experience the effects of cyberpower together. Additionally, ideas of dependency and paternalism can be revisited as mechanisms of power are redistributed, creating an environment conducive to collective self-mobilization capable of unsettling democracies, as everything has become dependent upon a system that: “… makes it easier to subvert and harder to govern” (Betz and Stevens, 2011, P. 135). As digital citizenry expands, the need for leadership and systems of governance with the capacity to operate in ways that mitigate the negative effects of cyberpower are necessary to support transformations in developed and less mature institutions in developing countries (Goodhand, 2006; Tapscott, 2014; Bellinger, 2016).

New tensions and conflicts created by the effects of cyberpower add uncertainty to processes of institutional development. For example; on the one hand, governments have to devolve: “…responsibilities and authority to private actors” (Nye, 2011, p. 16) in order to ensure state cyber security. This makes for fragmentation and inefficiency in operationalizing cyber security provision. On the other hand, governments are being accused of not taking enough responsibility to ensure citizens are correctly educated, or being told they need to do more to: “…ensure an efficient and unified approach…” (Norwegian Centre for Information Security [NorSIS], 2016, p. 79) to meet the challenges presented by cyber. Investigating amongst key stakeholders the processes, structures and capacities required to manage such tensions, uncertainties and emerging concepts can provide insights into achieving organizational collaborative capabilities (Huxham, 1993). This is consistent with the literature study that identified the need for greater collaboration, if managers hope to effectively control development in a digital society (Huxham, 1993; Nye, 2011; Tapscott, 2014; Hagen, 2016).

## Methodology

The purpose of the methodology was to open up conceptual boundaries and where possible build a more coherent understanding for the term cyberpower, and its real world effects. Where people don’t have knowledge due to lack of data or experience, this exploration can support understanding (Blackmore and Ison, 2007).

The levels of uncertainty surrounding the effects of cyberpower show how it is both a real and emerging challenge. This necessitated a principled investigation to ensure the researcher gained an evidence based understanding of the kinds of institutional and capacity constraints that restrict or facilitate different organizations to get things done – or not – within this environment of uncertainty. Establishing what is good or what is bad practice in uncertain institutional landscapes can become blurred when moral, empirical and ethical narratives vary. The intention of the methodology was to support an exploration of a shared, contested and emerging problem that is revealing itself as a dominant force over the developed and the developing.

The author purposely chose to sample individual stakeholder respondents from within seven sectors: Political, Military, Economic, Social, Infrastructure, Information, and Diplomatic as the focus of the analysis. This list is not exclusive but represents institutions that use cyberspace to: “create advantage and influence events” (Kuehl et al., 2009, p. 38) in Norwegian society, as well as internationally. The respondents were coded for in text referencing when citing evidence as follows: **PR** (political respondent: Senior member of Parliament); **DefR** (defense respondent**:** senior leader for military operations); **ER** (economic respondent: senior digital security engineer); **SR** (social respondent: international security & defense expert); **IR** (infrastructure respondent: head engineer for an energy directorate); **MR** (Media respondent**:** expert in media leadership and innovation); **DipR** (Diplomatic respondent**:** leader for digital strategy).

It was expected that the researcher would hear respondents presenting complex, different and emerging themes relating to cyberpower effects. Based on key themes that came out of defining and analyzing the problem, five categories were identified: *cyber governance, holistic approach, multi-stakeholder model, new approaches*, and *behavior of interested parties*. It was hoped that specific opinions and perspectives concerning these thematic areas would emerge.

To reflect the goals of the research, qualitative data gathered through semi-structured interviews was used as the primary method to inform about the development of operational strategies to manage cyberpower effects. The Overarching Questions (OQ) listed below in **Table 1** represent the main lines of enquiry. They were designed and applied to act as prompts, in order to allow for assessment and discussion within each of the five categories.

**Table 1:** Presents the overarching questions listed by category.

(1) Cyber governance
(1.1) What is understood by the term cyberpower?
(1.2) What do key stakeholders understand by the terms dependency and vulnerability in the context of the operation of cyberpower? In what ways are the terms seen as being in tension (or not)?
(2) Holistic approach
(2.1) In what ways are the tensions between vulnerability to and dependency upon cyberpower manifested in operational terms?
(3) Multi-stakeholder model
(3.1) What kinds of processes and structures could support managing the tension between vulnerability to, and dependency upon cyberpower?
(4) New approaches
(4.1) What might a bottom-up approach to managing the effects of cyberpower entail?
(5) Behavior of interested parties
(5.1) What kinds of institutional and capacity constraints work against effective collaboration/co-ordination between organizations, and how might those constraints be managed and negotiated?

*Overarching questions by category*.

The limited sample size means data is partial and only presents selective visibility of each sector (Mukherjee and Wuyts, 2014). However, deciding to secure subjective information from senior respondents as the primary data source helped the researcher learn from people who know within operational environments (Hanlon, 2014). A consequence of this method is possible bias and value laden data as: “…those in positions of power may also be in good positions to see and explain what is going on, even though their self-justifications are likely to be biased” (Thomas and Chataway, 2014, p. 333).

Given the emerging and non-boundaried nature of this problem (Woodhouse, 2014) the researcher was aware that organizational culture would frame language. This presented potential contestations based on partiality of knowledge consisting of multiple truths. When perceptions are being framed by the wider context within which they operate (Everitt, 1996), then questions arise relating to what each respondent considered and evaluated as good practice when managing cyberpower in a democratic society. This richness supported the choice of conceptual framework as it brought forward the complexity and emergence of a developing landscape around cyberpower. Additionally, the researcher needed to be aware that each organization is living with pressures exerted on them from the broader environment. These shape the organization’s actions and influence how they choose to respond to interview question (Roche, 2014).

Reflecting on the idea of shared responsibility (Thomas, 1996) highlights the fact that citizens have a participatory role in shaping and defining how institutions need to develop in response to cyberpower. However, concerns about data quality arising from the lack of problem knowledge amongst random samples meant the researcher decided not to interview members of the public. Not having this data leaves a gap in perspectives that would have contributed to the project. Especially considering the earlier definition of cyber security as protecting individual and societal wellbeing. This is certainly true in light of the current view that participatory – or what appears to be commonly referred to now as a holistic approach – is deemed the most appropriate for managing digital uncertainties (Tapscott, 2014; Hagen, 2016).

Respondents answered critically to interview questions as they reflected on the complex problem. This triggered the researcher to formatively question how their appreciation for the problem was developing. In almost all cases the respondent reported a change in understanding and perception. This shows how the line of questioning prompted focused reflection on this emergent theme, indicating that respondents were willing to embrace complexity and seek to understand it: “…rather than oversimplifying reality…” (Mayoux and Johnson, 2014, p. 186) or simply taking issues for granted.

The seven informants – two female/five male – are leading in a field they have not yet mastered, nor fully understood its true complexity; yet they act in, on and around this continually contested space every day (Goodhand, 2006).

This study was carried out in accordance with the recommendations of The Norwegian Data Protection Authority. All subjects gave written informed consent to be interviewed. The subjects have been anonymized, no sensitive personal information was collected and no data has been stored electronically.

## Results

Data was analyzed using the five thematic areas: cyber governance, holistic approach, multi-stakeholder model, new approaches and, behavior of interested parties. Within these categories a number of key sub-themes appeared. These sub-themes are presented in **Table 2** and will form the basis for discussion in the next section. The results analysis also looked for correlations and differences between respondents and the literature study. Attention is drawn to themes of variations in perspectives, complexity, uncertainty, and emergence.

**Table 2:** Lists the key sub themes that appeared during interviews.

Cyber governance	Holistic approach	Multi-stakeholder model	New approaches	Behavior of interested parties
Political role	Sharing	Best practice	Challenge institutional norms	Value conflicts
Influence	Informal collaborations	Conflicting interests	Human factors	Approach to the Sector-principle
Institutional uncertainty about how to govern	Organizational culture relating to risk	Systems of governance	Framed by uncertainty	Willingness to co-operate
Dependency vs. vulnerability		Power relations		Lack of trust
Complex intra-organizational relationships				Collaboration for survival

### Cyber Governance

Perspectives varied depending upon how different sectors experience or use cyberpower. One respondent stated that cyberpower is: “not cyberwar” (SR) contra DipR who considered cyberpower to be a term that describes cyber warfare. Another respondent bridged these two polarized perspectives by using the contemporary ‘Hybrid War’ concept to describe cyberpowers real world capacity to: “undermine and influence another states political authority” (PR).

As the examples above show, the *political role* of cyberpower emerged as a common theme among different respondents. Further, respondents commented that it can be used to: “gain power to influence for a purpose” (ER) and, it is how we: “influence people through different levels of power” (SR). For some this was a positive development as it: “protects values, people, info, property, reputation, and operational ability” (DipR). While for others: “it’s a difficult grey-zone” (DefR), and its role has more negative connotations: “using and modifying information technology for your own purpose” (ER). Also, and in line with the problem definition, it can be both: “Cyberpower is maybe the power to influence a target group, or a population, through cyber means; for personal objectives – good or bad” (IR).

Corresponding with the Kuehl et al. (2009) definition of cyberpower, all respondents, including those who had either not heard the term before, were unfamiliar with it or found it “comprehensive” (DefR), used the term *influence* when describing their understanding of cyberpower.

Further discussions revealed *uncertainty how to govern* based upon institutional understanding. All respondents were not surprised different organizations/respondents had different understandings of cyberpower. As one respondent explained: “organizations have different cultures and each is expected to look at cyber risk and threats differently” (IR). This can be related to political tensions around usage, i.e., the military uses the same national infrastructure that citizens do. Or as another commented: “cyber is creating an arena for political development” (PR).

Uncertainty was an emerging shared theme concerning small vulnerabilities that can present massive consequences. This uncertainty is driving immediate reactive practices (ER; DipR; IR) over long-term strategies. All respondents agreed that dealing with the volume of network vulnerabilities complexifies and negatively affects longer-term planning. For example in journalism, a consequence of the operation of cyberpower is: “the amount of uncertainty today means so much confusion looking into tomorrow” (MR).

Governance uncertainties were again revealed as dependency and vulnerability were reported to frame an uncertain institutional landscape as organizations may well act in ways that have consequences for social change, as traditional systems of governance and codes of practice are challenged (Stevens, 2015). As one respondent commented: “traditional means of protecting sovereignty lack the necessary control instruments to manage cyber effects” (DipR).

If the operation of cyberpower is creating the above context then a respondent opinion that attempts to resolve tensions from the *dependency vs. vulnerability* paradox will follow the same developmental path as the analog world did, presents an interesting discourse. The respondent stated: “People and their ideas have not changed […] we are not more honest, our intentions are no better, our wishes for intake and welfare do not change” (PR). The PR respondent followed this by saying mechanisms for managing the operation of cyberpower will: “become more dramatic” and “need to involve national digital borders” (PR).

Inevitable tensions for a small nation like Norway – that has physical territory but everything it does is dependent upon digital interactions with the international community – arise from the scenario presented by PR. These were emphasized by DefRs description of future tensions if the state controls Internet freedoms through digital borders and monitoring. This the respondent said: “threatens a human right, the economy and welfare security” (DefR).

Tensions are apparently being hidden as a result of vulnerability: “tensions don’t manifest, if they did, then actions would be taking place to address the problem” (SR). Soon after this interview Norway’s largest digital provider Telenor was accused of failing to take their responsibility to society seriously. The company apparently chooses not to report all digital-crime because they are afraid to loose control and do not wish to co-operate with the police. In response, Telenor stated they do not have time to review and report all incidents (Njie, 2017).

The DipR presented an interesting situation that adds complexity and highlights that difference in *intra-organizational relationships* affects institutional development processes. The respondent explained how one group within the Norwegian Foreign Office runs cyber business, i.e., daily operations. Whilst another group runs the international political policy face of cyber, rarely do the two groups meet to calibrate policy, practice or governance.

### Holistic Approach

As a member of the committee who established the national cyber incident reporting process, IR was insightful and un-surprisingly loyal to the system designed to be inclusive and ensure information *sharing* across sectors. The IR did acknowledge that the process is not universally applied or governed across sectors.

A comment by ER revealed how *informal collaborative* processes can take place beyond institutional frames. The ER explained how they communicate with online groups who analyze malicious malware, as a means of finding solutions to new vulnerabilities. Similarly, MR noted how journalists have to look elsewhere to find the truth, i.e., away from: “institutions of democracy such as the White House” (MR).

The ER formally worked in the Defense sector and highlighted how different *organizational cultures* shape how they respond to tensions. The ER described how availability is an exercise in accepting risk in the Norwegian economic sector. Meaning they prioritize network availability, such as BYOD or mobile banking. However, the Defense see this exercise in risk acceptance as unnecessary or not a priority.

### Multi-Stakeholder Model

The IR commented that sharing knowledge makes knowledge and power grow. This reflects the literature that calls for increased power and understanding through sharing of information beyond institutionalized boundaries (Thomas, 1996; Castells, 2011; Nye, 2011; Tapscott, 2014). Interestingly, IR followed this statement with skepticism toward cross sector collaboration. In IRs view, the core business of cyberpower for the military is different to other sectors and: “It’s not necessarily true that if you are good in one domain you can be good in another” (IR). It was unclear if this was an organizational culture issue or a question of *best practice* relating to skills and capacities not matching.

Similarly, MR stated that journalistic organizations are working hard to develop ethical standards and new institutional best practices in response to cyberpower effects such as online fake news. However, they are unwilling to coordinate outside of their sector for fear of diminishing the integrity of the journalistic profession.

The ER and DipR called for more government regulation to: “address concern and confusion about what is going on in cyberspace” (ER). SR acknowledged some top-down efforts were in place, however, complexity arises due to *conflicting interests* as: “everyone has different premises and therefore how do you know the model is correct” (SR). Meaning theoretically: “Things will never be fine, we are always on our way to an improved state” (IR). Or the problem is so complex and emerging, that finding/negotiating an ethical balance between dependency and vulnerability is severely contested when authorities: “start watching what you are doing on the net […] then the state becomes something else” (DefR). This is significant when compared to comments about mechanisms for managing cyberpower made by PR in section 4.1 Cyber Governance.

There were differences in perspectives regarding *systems of governance*. PR judged a digital society needed a political system that was up-to-date with the challenges, and Norway: “being small and democratic should be OK” (PR). In contrast, DefR questioned how the Norwegian democratic system is going to cope with big emerging challenges expedited by cyberpower that see voters seeking: “less uncertainty, less integration, and less globalization” (DefR).

If each sector develops independently with its own values and cultural dimension, cultivating: “truths of practice” (Everitt, 1996, p. 179) in relation to managing vulnerabilities. Then they are able to justify (in theory) their unique role in contributing to a just, open, safe, and secure society. As SR commented: “The problems are not new, they are just more interconnected. We need to identify what is different” (SR). However, without a common platform for collaboration the status quo - represented by *power relations* framed in the sector-principle – is not challenged. This is significant when institutional development depends upon the level and sum of relationships between people and organizations (The Open University, 2007).

### New Approaches

All respondents agreed that leadership and generational factors have a role in managing the effects of cyberpower. The DefR called for new leadership philosophies that mirror technological solutions and reach beyond a: “fenced in physical domain” (DefR). This was explained as leadership driven by capabilities and creating effects in wider networks. Moving from leadership being simply about position, ownership and power, to styles that operate with bigger pictures in mind. This concept echoes with the idea of knowledge sharing making power grow (see IR, section “Multi-Stakeholder Model”). These ideas present a value-based conflict as they *challenge institutional norms* for those currently occupying senior positions. As they imply reluinquishing forms of power.

Considering the above, with regards to preparing the next generation of leaders/managers, implies the requirement for scaffolding innate technical skills and encouraging flexible cognitive strategies for operating in the new digital economy (Hoffman and Hancock, 2017; Knox et al., 2017). The PR respondent gave an opinion that presents a challenge to these ideas when describing a real world context: “The younger generation is more segmented and have no need for broad perspectives” (PR). The PR explained that this has a negative/weakening effect on democratic development as it can lead to political polarization and a less dynamic political debate. These contrasting views emphasize the uncertainty surrounding *human factors* in digital societies.

The PR and IR respondents commented that state aparatus such as banks, critical infrastructure and democratic process are being threatened in: “new ways” (PR); leading to increased political responsibilty to manage it. This can be seen as a call for new approaches to managing cyberpower effects that require *new literacies* (Kellner, 2002) at political governance level. Another respondent noted that a vulnerability arising from mass-media is that people: “…are not skilled enough to know how to trust it” (MR). New literacies identified in the literature study are those that mirror the digitally interactive environemt todays students have grown up in and have the potential to answer the question: “where does trust come from?” (Harriss, 2000). As a respondent noted: “Leadership today is more about the kind of person you are not the kind of age you are” (IR).

### Behavior of Interested Parties

Individual and organizational *value conflicts* surfaced that create uncertainty and tension for effective collaboration. Suprisingly, little was presented to indicate longer term solutions that go beyond institutional frames (The Open University, 2000) to manage these tensions. Norway is rapidly digitalizing as a condition of a modern society (Stortingmelding, 2016–2017); and by doing so, is knowingly increasing vulnerability to negative development (Jasper, 2012). A respondent made this point clear: “we are pushed to use the cyber domain” [and establish a] “shared security culture within a domain that is inherently vulnerable” (DipR).

It would appear that *approaches to the sector-principle* may be leading to negative developments in the face of cyberpower challenges. This may be due to multiple conflicting formal and informal policy and practice agendas shaping progress. The sector-principle was described as: “good, but a big challenge” (DefR). Further, three constraining factors were presented; “It’s very hard to coodrinate cross sector as each sector has its own Department; threats in one sector may not get heard by another; the principle makes it hard for effective use of time and resources” (DefR). These conditions are compounded by powerful unwritten rules that incentivize or sanction depending on different interpretations (Brett, 2000). One such example is: “When it comes cyber, its effects and managing collaborations, industry is the one leading the way and setting the agenda” (IR).

The *willingness to co-operate* across sectors is restricted for the exact reason that: “…it implies reciprocal sharing of rights and responsibilities” (Robinson et al., 2000, p. 226). When cyberpower is added to this context, with its power to out pace traditional good governance and codes of practice (Stevens, 2015), then a conflicted institutional development landscape is manifest. If interested parties want to develop, then it: “may require a new way of collaborating” (DipR) as conventional models of sharing/information exchange may no longer apply. However, when generalizing about current behaviors, a respondent commented: “You can build a network but it doesn’t exist if it is not used” (PR).

In a time when *lack of trust* – due to multiple vulnerabilities – is a major factor shaping action, all respondents believed there is a will to collaborate and co-ordinate to improve management of vulnerabilities arriving through cyberspace. However, when each sector investigated; political, defense, economic, diplomatic, infrastructure, media and social has its own policies, domain of responsibility and private sector service providers; then the idea of co-operating with another sector – that has a completely different set of operating conditions and core business relating to how it applies or manages cyberpower – was seen as something that would create tension (IR; MR).

The MR gave an example of *collaboration for survival*. In the financial sector banks are collaborating to build power in the area of digital payment via mobile apps. This way smaller banks gain strength, and improve their survivability against future ‘online’ – international – competitors (Reuters, 2017). In the media sector: ‘no option’ collaboration was described as ‘convergence’, and exemplified by the amount of mixed content online (MR). The MR commented that agreeing the ethics of media and journalism when cyberpower is re-shaping the core of the industry is a major persistent challenge. This is a consequence when the rules of the game are changing (North, 1990) and tensions lead to: “imperfect compromises” (Nye, 2011, p. 16).

## Discussion

The following evidence-based discussion relates empirical findings to the characteristics of the problem. The intent is to nudge thinking to avoid taken-for-granted practices that may be blocking institutional development (Everitt, 1996) and where possible; open the debate among stakeholders concerning the complexity, difference and emerging ways cyberpower is impacting institutional development.

The different cross sector perspectives revealed by respondents contribute to explaining why cyberpower is not yet a fully understood term. As the results reveal, cyberpower was shown to influence organizations differently depending upon their domain of interest. Significantly, the results not only align with the literature, they extend our understanding regarding the ubiquity of cyberpower. Critically the way cyberpower affects institutional development, across all levels of society, make its role inherently political (Standt, 1991) and therefore a governance challenge. The complex characteristics of cyberpower – most prominently seen as emerging positive uses and negative abuses – ranging from those that enrich human life through access to information, to those that undermine another land’s political authority; imply the need for a wider political space if there is to be a common platform for collaboration (Wood, 2003). Taking a shared responsibility (Thomas, 1996) approach to cyber security – in the form of jointly developed national strategies and guidelines (Helkala and Svendsen, 2014) – could present opportunities for improving certainty around governance policy and practice. This may in turn encourage better intra and inter-organizational relationships for institutional development.

The finding that one respondent found cyberpower a difficult term (IR), another didn’t use it (PR), and one viewed it as comprehensive (DefR), is a valuable outcome. Not only does it emphasize the richness of the problem, the fact that respondents were able to locate themselves and their organization within the problem space, means it has some level of relevance for them. Going forward, defining cyberpower in a way that demands less explicit knowledge of the term ‘cyberspace’ and less effort to unpack the slightly abstract terms: ‘operational environments’ and ‘instruments of power’ (Kuehl et al., 2009, p. 38) could make the concept more approachable. In practical terms this would help penetrate the consciousness of a wider demographic by situating the word *cyberpower* into their everyday lexicon. This would support moving understanding forward from the current state where cyberpower has relevance, but conflicting interests and value conflicts mean it is not yet a well categorized or operationalized concept.

The interview responses from ER, DipR and IR build on the understanding that organizations are relying on individualized assets to seek immediate security priorities ahead of pursuing long-term goals (Wood, 2003). If this deliberate survival strategy response to dealing with uncertainty reflects organizational best practice in digitally advanced Norway; then the likelihood is that governance uncertainties in developing states – with weaker institutions and governance mechanisms – could be more of destructive and destabilize their digital moral universe (Wood, 2003; UNWOMEN, 2015). The operation of cyberpower is increasing vulnerability and creating consequences for dependency that present a new ‘Faustian bargain’: staying dependent, staying vulnerable. This occurs as the negative effects appear to frame: “dysfunctional time preference behavior” (Wood, 2003, p. 455). As reported by ER, DipR, IR, and MR, institutional governance uncertainty when dealing with immediate vulnerabilities and insecurities arriving through cyberspace, displaces individuals and organizations ability to focus on long-term strategies (Wood, 2003).

The near horizon debate in Norway concerning digital borders will be framed by governance uncertainty and could have destabilizing effects as it concerns not just: “human rights, the economy and welfare security” (DefR). It will also influence organizational cultures, environmental factors and power dynamics between government, the private sector and citizens. Institution leaders need to learn from these tensions and share knowledge gained, good and bad. This is necessary to support longer-term strategies designed cope with cyberpower effects. A number of respondents described the non-collaborative practices of interested parties and unwillingness to share. When attempting to manage the effects of cyberpower, such issues should be addressed, and institutional building should be seen as: “…an incremental, sequential process which depends on learning and stimulates self-transformation” (Harriss, 2000, p. 234). Meaning that collaboration, in the form of information sharing, can form the foundation of co-operation based upon communication, reframing of problems and shared learning. If building co-operation is a step to institution building (Harriss, 2000), then the results of this research reveal the pressing need for new approaches that build collaboration capacities capable of facilitating better co-operation over time.

Institutionalized systems of governance and practices framed in policies: “crafted to meet the needs of the past” (Tapscott, 2014, p. 390) are struggling to: “get ahead of the game for long enough to really commit resources for the future” (Wood, 2003, p. 458). This may be the case in Norway as national governance structures limit the prospect of open collaboration in the face of emerging vulnerabilities arising from digital dependency. The comments by DefR that the sector-principle is a big challenge and makes it hard for effective use of time and resources counter the assumption that the sector-principle and a multi-stakeholder approach to cyber security form any kind of best practice (Carr, 2015), or solid enough basis for organizational linkages (Salancik and Pfeffer, 1978; in Thomas, 1996). Any delay or failure to adapt to a problem that is redefining institutional rules of the game in society (North, 1990; Tapscott, 2014) will only increase transaction costs in the long term (Ouchi, 1980).

As the results indicate, the effects of cyberpower are creating an institutional landscape framed by leadership uncertainty and a complex struggle about what is: “getting the work done by the best means available” (Thomas, 1996, p. 100). A consequence of this may be more government control (Helkala and Svendsen, 2014; Tapscott, 2014; Norwegian Centre for Information Security [NorSIS], 2016) resulting in top-down command and control mechanisms (Fayol, 1916/1949). For the respondents in this research, this development may represent a ‘more dramatic’ governance mechanism (PR), the idea of the state becoming something different than it is today (DefR), or an approach that may falter due to conflicting interests concerning models of governance (SR). In Norway, this praxis would run against cultural norms and meet significant resistance. Therefore, maintaining the status quo and pursuing the management policy of empowering and enabling (Paton, 1991) sectors – that is “molded to the interests of […] the state” (Thomas, 1996, p. 104) – will require greater space for expressive innovative collaborations if Norway is going to manage the tensions between vulnerability to, and dependency upon cyberpower.

The problem definition identified that managing cyberpower may require new and more adaptive approaches beyond current institutional frames (Conklin, 2006; McDonnell, 2016). The results build on this as respondents identified new human factor capability based leadership models that create effects in the wider network (DefR), and are less about age and more about personality and knowledge (IR). Further, when Castells (2011) and Tapscott (2014, p. 89) refer to building relationship capital through a collaborative infrastructure, then the above approaches may well support prosperity through the practice of trust, and joint decision-making by providing the: “cultural glue [for the] …new [digital] developmental paradigm” (Castells, 2011, p. 213).

New literacies that integrate conceptual and skill areas (Thomas, 1996, p. 100) are those that include critical thinking, complex problem-solving, expert communication and applied knowledge in real world settings (Peña-López, 2016). At a personal and organizational level this implies the need to develop a range of capacities capable of building the relationship capital necessary for managing engagement with the digital world.

The interview data highlighted that modes of governance for managing cyberspace – and the vulnerabilities it presents to public and private entities – could lie beyond the current sector-principle institutional frame. Power dynamics between sectors (Muller, 2016) need to become more than barriers to comprehensive cyber security. Power needs to become the catalyst for development of new knowledge through improved collaborative behavior. All respondents stated the need for better organizational strategies, a greater capacity to find long-term solutions to novel vulnerabilities, and identified the understand function as a weakness across sectors. Choosing *not* to manage the dependency vs. vulnerability paradox could be a source of failure as the negative effects of cyberpower lead to institutional decay.

## Conclusion

We are witnessing powerful effects arriving through cyberspace that present real world shared problems that could not have been foreseen. A consequence of this is the political reality that existing national structures of governance may be struggling to cope. When a functioning society and all its assets – tangible and intangible – depend upon cyberspace then a holistic approach explicitly frames all parts of the whole (OED, 2017). This means that cyberpower and its unpredictable impacts on social change processes (Thomas, 1996) demands greater attention.

Uncertainties emerging from cyberspace will persist as technology develops, governments support digital rollout for economic development, and increased network capabilities lead to planned positive outcomes and, emerging negative outcomes for people and organizations dependent upon cyberspace. Vulnerabilities are leading to time dysfunctional behavior and possible destructive decision-making in the form of for example imposing digital borders. The implication for institutional development is how to create the necessary national trust conditions across and within sectors that encourage openness, transparency and information sharing for positive institutional development in times of uncertainty.

The idea that understanding is no longer a condition of knowing but an activity involving dialog (Everitt, 1996) frames the current situation regarding man-made cyberspace. Dealing with emerging challenges and: “fundamental changes to society” (Tapscott, 2014, p. 386) may require dialogical activities that realign institutional frames to ensure what we created – but no longer understand (Nye, 2011) – does not undermine institutional development processes. As the data from this study reveals, if sectors and organizations are not mandated to follow national cyber incident reporting structures, or are not: “directly subject to a national cyber security policy” (ER); then this demonstrates why leaders need the capacities to work with uncertainty, to negotiate complex institutional landscapes and relationships, understand threats and identify opportunities to bring stakeholders together.

The extent to which the respondents showed an awareness of the need for these capacities within their organization was inconclusive. What is apparent is that advancing institutional agendas in dynamic environments, where outcomes cannot be predicted is more about maintaing current operational output, than investing in human capacities capable of providing longer-term development solutions.

As one respondent noted:

• The shared perspective concerning cyberpower is that people are reluctant to look too closely at the problem cos they don’t know the right questions to ask if they do, and they lack the imagination about what might come or be about to happen. They are reluctant to accept their own vulnerability (DipR).

Co-operative processes can reveal organizational common ground and interdependencies as opposed to stressing the differences (Robinson et al., 2000). Where collaboration potential exists beyond organizational boundaries (The Open University, 2007), then a new model of collaboration that activates engagement between stakeholders can become a key feature to support shared understanding, founded on common values and goals. Driven by the need to build capacities capable of managing joint vulnerabilities that arrive through cyberspace and contribute to decay or weakening of institutions (Robinson et al., 2000).

The governance challenge, when policies and practices are institutionalized, is apparent in Norway. Transforming the sector-principles’ capability to function in a digital development paradigm means moving from a regime of truth that maintains power relations in Norwegian society (Everitt, 1996) to a model capable of responding to the new approaches, opportunities and challenges posed by cyberpower. This study has shown that this will require management of conflicting values, novel developments and emerging contexts, founded on capacitates capable of mobilizing resources and shaping alliances (Thomas, 1996).

Recommending practices that can become the strategic corner stone for managing the ways institutional development is affected by the growing influence of cyberpower – such as the dependency vs. vulnerability conflict – will involve new models of leadership that educate and empower-to-enable younger generations to operate beyond structural frames. This will allow for collaborative advantage in ways that are context orientated rather than restricted by hierarchical norms of communication structures (Knox et al., 2018). This means individuals being free to deliver effects in the broader environment beyond organizational boundaries in collaboration with trusted partners.

To conclude, the following recommendations are made:

(a) Further research aimed at defining cyberpower in a way that is more universally appealing and usable. For example:
Cyberpower is the capability to influence tangible and intangible assets through digital means.
(b) Organizations should commit time and human resources to improve institutional development prospects when faced with effects of cyberpower. Not doing so means continuing to contribute to their own vulnerability leaving them more precarious as they: “stretch[…] out a survival strategy” (Wood, 2003, p. 229).
(c) Conceptualize cyberpower as a whole inter-organizational domain of shared responsibility to improve prospects of achieving a holistic approach.
(d) Encourage the merging of inter-organizational boundaries enabling new relationships – grounded in shared responsibilities – to emerge, that can transform management models and strategies.
(e) Encourage expressive innovative collaborations oriented toward co-creation and: “building up the capacity to maintain influence into the future” (Thomas, 1996, p. 103).
(f) See managing cyberpower as a shared development: “activity and attitude” (Wrangham, 2016) founded upon skills such as unstructured problem solving, critical thinking, learning and reasoning. To build these capacities takes modes of education that focus on: “non-routine, higher order cognitive skills”, relating to: “new [digital] economy skills” (Peña-López, 2016, pp. 123, 267).

## Future Work

Cyberpower is creating a context where a lack understanding about tomorrows threats, leads to fragmentation, inefficiency and actions that: “favor meeting immediate needs over future ones” (Wood, 2003, p. 231). Firstly, bringing stakeholders together – who would not normally interact over this problem – would support a more principled investigation in terms of relationships between actors. Action research of this type would be a valuable next step, as the outcomes may support more evidence-based recommendations for managing collaborations intended to advance institutional agendas in a dynamic environment where outcomes cannot be predicted. Secondly, to add depth and richer comparative evaluations, further data collection from within individual sectors could reveal internal data patterns, as well as allow for increased comparison across sectors at respective hierarchies.

This research contributes empirical data to an emerging and globally shared phenomenon that is: “…posing more questions than answers” (Tapscott, 2014). Through seeking to ground the term *cyberpower* in the lexicon of change agents through a process of interweaving different perspectives and understanding, this study and related future work could broaden the range of conceptual tools available to development mangers when addressing the issues of dependency vs. vulnerability.

## Author Contributions

BK confirms being the sole contributor of this work and approved it for publication.

## Conflict of Interest Statement

The author declares that the research was conducted in the absence of any commercial or financial relationships that could be construed as a potential conflict of interest. The reviewer BH and handling Editor declared their shared affiliation.
